# Tailored biosynthesis of diosmin through reconstitution of the flavonoid pathway in *Nicotiana benthamiana*

**DOI:** 10.3389/fpls.2024.1464877

**Published:** 2024-10-18

**Authors:** Hyo Lee, Sangkyu Park, Saet Buyl Lee, Jaeeun Song, Tae-Hwan Kim, Beom-Gi Kim

**Affiliations:** ^1^ Metabolic Engineering Division, National Institute of Agricultural Sciences, Rural Development Administration, Jeonju, Republic of Korea; ^2^ Department of Animal Science, Institute of Agricultural Science and Technology, College of Agriculture and Life Science, Chonnam National University, Gwangju, Republic of Korea

**Keywords:** diosmin, diosmetin, flavonoid, synthetic biology, transient expression, *Nicotiana benthamiana*

## Abstract

The flavonoid diosmin (diosmetin 7-*O*-rutinoside) is used as a therapeutic agent for disorders of the blood vessels such as hemorrhoids and varicose veins. Diosmin is commercially produced using semi-synthetic methods involving the oxidation of hesperidin, the most abundant flavonoid in citrus fruits. However, this method produces byproducts that are toxic to the environment, and new sustainable methods to produce diosmin are required. Here, we used a synthetic biology approach to produce diosmin without generating toxic byproducts through reconstitution of the diosmin biosynthetic pathway in *Nicotiana benthamiana*. We first established that *N. benthamiana* leaves co-infiltrated with all seven genes in the flavonoid biosynthesis pathway produced high levels of luteolin, a precursor of diosmetin. We then compared the activity of modification enzymes such as methyltransferases, glucosyltransferases, and rhamnosyltransferases in *Escherichia coli* and *in planta* and selected genes encoding enzymes with the highest activity for producing diosmetin, diosmetin 7-*O*-glucoside, and diosmin, respectively. Finally, we reconstructed the entire diosmin biosynthetic pathway using three constructs containing ten genes encoding enzymes in this pathway, from phenylalanine ammonia lyase to rhamnosyltransferase. *N. benthamiana* leaves transiently co-expressing all these genes yielded 37.7 µg diosmin per gram fresh weight. To our knowledge, this is the first report of diosmin production in a heterologous plant system without the supply of a precursor. Successful production of diosmin in *N. benthamiana* opens new avenues for producing other commercially important flavonoids using similar platforms.

## Introduction

Diosmin (diosmetin 7-*O*-rutinoside), a natural flavone glycoside of the aglycone diosmetin (3′,5,7-trihydroxy-4′-methoxyflavone), was first isolated in 1925 from figwort (*Scrophularia nodosa* L.). Given its ability to mitigate venous insufficiency, diminish inflammation, and restore normal blood flow, diosmin has long been used as a phlebotonic and vascular protector (Ahmed et al., 2016; Abdel-Reheim et al., 2017; Feldo et al., 2018; Shalkami et al., 2018; Huwait and Mobashir, 2022). Furthermore, various *in vitro* and *in vivo* studies have indicated that diosmin has antioxidant, anticancer, antidiabetic, and mild antibacterial activities (Pietrzycka et al., 2015; Lewinska et al., 2015; Mustafa et al., 2022). The demand for diosmin in the nutraceutical and pharmaceutical markets is increasing rapidly.

Diosmin is produced by citrus plants and its abundance varies based on the species, tissue type, and maturity of the fruits, with immature fruits usually having higher concentrations of diosmin. However, in various citrus plants (e.g., *Citrus limon*, *C. aurantium* L., *C. limonia* Osbeck, *C. medica* L., *C. reticulata* Blanco, and *C. sinensis* Osbeck), the concentration of hesperidin, which can be converted into diosmin, in citrus peel (400–23,000 µg/g dry weight [DW]) is higher than that of diosmin (0–440 µg/g DW), and diosmin is mostly absent in the pulp (Chen et al., 2020; Liu et al., 2021). Therefore, diosmin has been commercially produced from hesperidin using a semi-synthetic method involving the oxidation of hesperidin extracted from citrus fruits. However, this method produces byproducts that are damaging to the environment and requires additional clean-up steps and supply of raw materials is unstable (Zhao, 2022). Thus, several efforts have been made to produce diosmin directly in a biological system. Wang et al. (2021a) produced diosmetin from hesperitin by expressing the gene encoding flavone synthase (FNS), an enzyme in the flavonoid biosynthetic pathway, in *E. coli*. In addition, Deng et al. (2024) produced diosmin *in vitro* through a multi-enzyme cascade reaction involving CiCOM10 (methyltransferase), CiUGT11 (glucosyltransferase), and CiRhaT (rhamnosyltransferase) derived from *Chrysanthemum indicum*. In this system, luteolin, an upstream metabolite of diosmin, was provided as the substrate.

**Figure 1:**
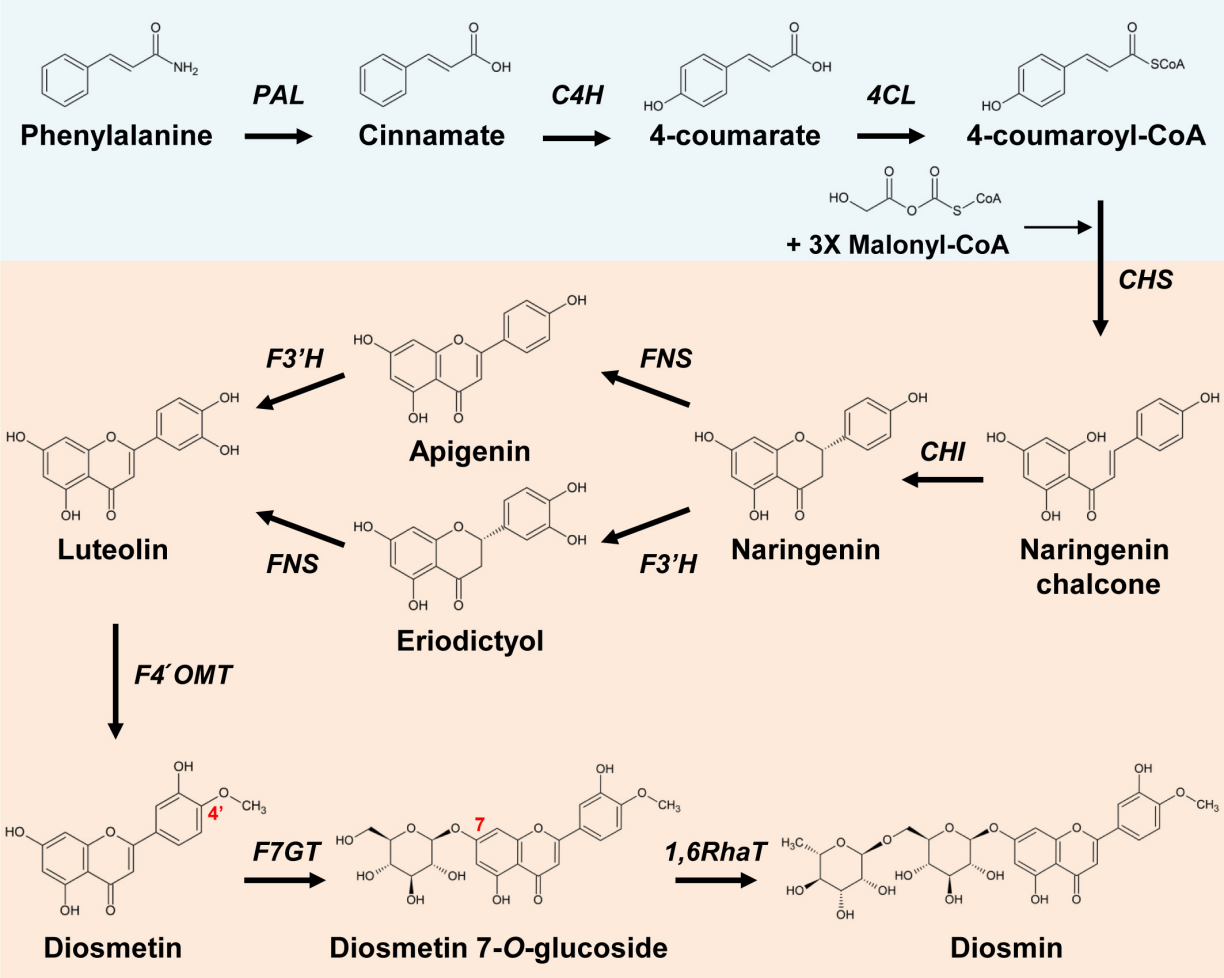
Proposed diosmin biosynthetic pathway from phenylalanine. The phenylpropanoid pathway (blue box) starts with phenylalaine produced by the shikimate pathway. The diosmin (red box) biosynthesis pathway starts with chalcone synthase (CHS) converting three molecules of malonyl-CoA and one molecule of 4-coumaroyl-CoA into naringenin chalcone. PAL, phenylalanine ammonia lyase; C4H, cinnamate 4-hydroxylase; 4CL, 4-coumarate ligase; CHS, chalcone synthase; CHI, chalcone isomerase; FNS, flavone synthase; F3'H, flavonoid 3'-hydroxylase; F4′OMT, flavonoid 4′-*O*-methyltransferase; F7GT, flavonoid 7-*O*-glucosyltransferase; 1,6RhaT, 1,6-rhamnosyltransferase.

Luteolin is biosynthesized through the phenylpropanoid pathway, which consists of phenylalanine ammonia lyase (PAL), cinnamate 4-hydroxylase (C4H), and 4-coumarate ligase (4CL), and from the flavonoid biosynthetic pathway, which comprises chalcone synthase (CHS), chalcone isomerase (CHI), FNS, and flavonoid 3′-hydroxylase (F3′H) (**Figure 1**) (Ferrer et al., 2008; Muruganathan et al., 2022). 4′-*O*-methylation of luteolin produces diosmetin, the aglycone of diosmin; the two-step glycosylation of diosmetin by 7*-O-*glucosyltransferase and 1,6-rhamnosyltransferase (1,6RhaT) completes diosmin biosynthesis (Tiwari et al., 2016). Several genes encoding methyltransferases that catalyze the methylation of flavonoids at various sites have been characterized from different plant species. Flavonoid 3′ or 3′,5′-*O*-methyltransferases that catalyze methylation at the 3′ or 3′/5′ position of the B ring include AtOMT1 (3′, *Arabidopsis thaliana*), CaOMT1 (3′, *Chrysosplenium americanum*), MpOMT3 (3′, *Mentha piperita*), SIMOMT4 (3′, *Solanum lycopersicum*) OsOMT1 (3′, *Oryza sativa*), ZmOMT1 (3′/5′, *Zea mays*), HvOMT1 (3′/5′, *Hordeum vulgare*), TaOMT1 (3′/4′/5′, *Triticum aestivum*), and CrOMT2 (3′/5′/7, *Citrus reticulata*) (Liu et al., 2020). *PaF4′OMT* (*Plagiochasma appendiculatum*), *MpOMT4* (*Mentha × piperita*), and *SOMT2* (*Glycine max*) have been identified as genes encoding flavonoid 4′-*O*-methyltransferases (Kim et al., 2005; Liu et al., 2017; Liu et al., 2022). Although citrus can be expected to have high 4′OMT activity, so far, only CreOMT1 and CreOMT4 (from *C. reticulata*) are highly associated with polymethoxyflavone (PMF) biosynthesis (Zohra et al., 2020; Liao et al., 2023).

Glycosylation by uridine diphosphate glucosyltransferases (UDP-glucosyltransferases, UGTs) is a common modification, and flavonoids are stored as glucosides in plants (Kim et al., 2006). UGTs can be divided into several groups, such as 3-*O*-glucosyltransferases, 5-*O*-glucosyltransferases, and 7-*O*-glucosyltransferases, depending on their regioselectivities (Zhao et al., 2024). Diosmetin 7-*O*-glucoside is biosynthesized from diosmetin by flavonoid 7-*O*-glucosyltransferase (F7GT). In *C. sinensis* (sweet orange), CsUGT76F1 converts diosmetin to diosmetin 7-*O*-glucoside and shows broad substrate specificity toward flavonoids such as hesperitin, naringenin, diosmetin, quercetin, and kaempferol *in vitro* (Liu et al., 2018). CiUGT11 (UGT88B3) was recently characterized from *C. indicum* and shown to glycosylate diosmetin to form diosmetin 7-*O*-glucoside; this enzyme displays wide-ranging substrate acceptance toward flavonoids such as apigenin, acacetin, luteolin, hesperitin, kaempferol, quercetin, and genistein at different positions (Deng et al., 2024). Diosmin is produced from diosmetin 7-*O*-glucoside by 1,6-rhamnosyltransferase (1,6RhaT) (Frydman et al., 2013). Cs1.6RhaT (*C. sinensis*) shows broad substrate acceptance toward the glycosides of flavonoid substrates (e.g., flavanones, flavones, flavonols, and anthocyanins) and catalyzes the rhamnosylation of flavonoid 3- or 7-*O*-glucoside (Frydman et al., 2013). Overexpression of *CiRhaT* (*C. indicum*) increase affects linarin, isorhoifolin, and diosmin production; this enzyme shows wide substrate specificity but preferentially uses acacetin 7-*O*-glucoside as substrate (Wu et al., 2022).

Biomolecules, such as high-value metabolites, can be biosynthesized in plants or other living systems by reconstituting their biosynthetic pathways using synthetic biology technology (Wang and Demirer, 2023). Plant systems are better suited for the production of secondary metabolites than are microbial systems, due to their cost-effective use of light and water (Barnum et al., 2021), their multiple cellular compartments and tissue types (Zurbriggen et al., 2012), and their possession of enzymes such as cytochrome P450s (CYPs) (Poborsky et al., 2023). *Nicotiana benthamiana* has been widely used as a model system in which to reconstitute natural plant product pathways, because it can transiently express foreign genes by *Agrobacterium* mediated infiltration at high efficiency, has a large biomass, and grows rapidly (Ma et al., 2003; Reed and Osbourn, 2018; Norkunas et al., 2018). The expression of multiple genes to reconstitute biosynthetic pathways in *N. benthamiana* has allowed the production of several natural products, such as lignans (Lau and Sattely, 2015), betalains (Polturak et al., 2016), etoposide (Schultz et al., 2019), strictosidine (Dudley et al., 2022), and baccatin III (Jiang et al., 2024).

In this study, we successfully produced diosmin through reconstituting its biosynthetic pathway in *N. benthamiana* leaves without providing substrate. Expression of all ten genes encoding enzymes in the diosmin biosynthetic pathway (from *PAL* to *RhaT*), divided into three optimized modules, resulted in the production of up to 37.7 µg diosmin per gram fresh weight (FW). This study sets the stage for producing other commercially important flavonoids in plant systems.

## Materials and methods

### Cloning of diosmin biosynthetic genes from different plant sources

Ten genes in the diosmin biosynthetic pathway were selected from different plants and used for module assembly: *PAL*, *C4H*, *4CL*, *CHS*, *CHI, FNS*, *F3′H*, *F4′OMT*, *F7GT*, and *1,6RhaT* (**Supplementary Table 1**). Type IIS restriction enzyme sites (e.g., *Bsa*I and *Bpi*I) within the genes were mutated to remove restriction sites without introducing changes in the encoded amino acids. The coding sequences (CDSs) of nine genes (*AtPAL*, *AtC4H*, *Sh4CL*, *OsCHS*, *OsFNS*, *OsF3′H*, *PaF4′OMT*, *CsUGT76F1*, and *Cs1,6RhaT*) were synthesized by Bionics Inc. (Korea). Four genes (*AtC4H*, *OsCHS*, *OsFNS*, and *OsF3*′*H*) were codon-optimized for efficient expression in *N. benthamiana* plants using the GenScript GenSmart™ Codon Optimization tool. The CDS of *BrCHI* was isolated from total RNA extracted from *Brassica rapa* L. leaves by reverse-transcription PCR using amfiRivert cDNA Synthesis Mastermix (GenDEPOT, Barker, TX, USA), PrimeSTAR HS DNA Polymerase (Takara bio Inc., Japan), and gene-specific primers (**Supplementary Table 1**) under the following conditions: 98°C for 2 min; 33 cycles of 98°C for 10 s, 58°C for 15 s, and 72°C for 1 min; and a final extension at 72°C for 3 min. The PCR products were purified using a QIAquickGEL Extraction kit (Qiagen, Germany). The ten genes (*AtPAL*, *AtC4H*, *Sh4CL*, *OsCHS*, *OsFNS*, *OsF3′H*, *PaF4′OMT*, *CsUGT76F1*, and *Cs1,6RhaT*) were subcloned into the level 0 (plCH41308) module using a Modular Cloning (MoClo) system (Addgene, USA) (Weber et al., 2011; Engler et al., 2014; Gantner et al., 2018) and verified by Sanger sequencing (**Supplementary Table 1**).

Three types of modules (level 0, basic modules; level 1, transcription units; level M, multigene constructs) were constructed using the MoClo system (MoClo Tool Kit #1000000044; MoClo Plant Parts Kit# 1000000047; MoClo Plant Parts II and Infrastructure Kit #1000000135). Transcriptional units (level 1 modules) were constructed by assembling the level 0 (Promoter, CDS, Terminator) modules (**Supplementary Tables 2**, **3**). Level M modules [PCFF (P, *AtPAL*; C, *OsCHS*; F, *OsFNS*; F, *OsF3′H*), PC4 (P, *AtPAL*; C, *AtC4H*; 4, *Sh4CL*), CCFF (C, *OsCHS*; C, *BrCHI*; F, *OsFNS*; F, *OsF3′H*), MGR (M, *PaF4′OMT*; G, *F7GT*; R, *Cs1,6RhaT*)] were constructed using the level 1 modules and an end-linker to build multigene expression vectors. Golden Gate reaction mixtures were prepared as follows: 40 fmol of each DNA insert or plasmid, 1.5 μl of T4 DNA ligase (400 U/μl, NEB), 1 μl of restriction enzyme, 2.5 μl of 10× ligation buffer, and sterile water to bring the final volume to 25 μl. *Bsa*I-HF v2 (NEB, USA) or *Bpi*I (Thermofisher, USA) type IIS restriction enzymes were used to assemble modules (Weber et al., 2011). After incubation in a thermal cycler, *Escherichia coli* was transformed with the entire reaction, and the resulting transformed bacteria were plated onto LB medium with the appropriate antibiotics and X-gal. White colonies were resuspended in 20 μl of sterile water, and 5 μl of the suspension was used for colony PCR (Marillonnet and Grützner, 2020).

### Substrate feeding assays of recombinant F4′OMT and 1,6RhaT proteins in *E coli*

Eight genes encoding F4′OMT and 1,6RhaT modification enzymes in the diosmin biosynthetic pathway were selected from various plants and synthesized by Bionics Inc. (Korea): *PaF4′OMT*, *MpOMT4*, *SOMT2*, *CreOMT1*, *CreOMT4*, *Cs1,6RhaT*, and two *CiRhaT*s (**Supplementary Table 1**). The CDSs of synthesized *F4′OMT*s (*PaF4′OMT*, *MpOMT4*, *SOMT2*, *CreOMT1*, *CreOMT4*) and *1,6RhaT*s (*Cs1,6RhaT*, *CiRhaT-GD4x*, *CiRhaT-AH2x*) were subcloned into the pENTR-D-TOPO vector using a TOPO cloning kit (Thermo Fisher Scientific, USA) and verified by Sanger sequencing. The CDSs of *F4′OMT* and *1,6RhaT* genes were cloned into the pGEX-6P-1 vector linearized by *Bam*HI digestion using an In-fusion Advantage PCR cloning Kit (Clontech, USA) and verified by Sanger sequencing; they were then transformed into *E. coli* BL21 (DE3) (Novagen, Germany). The transformed *E. coli* BL21 cells were grown overnight in LB medium with ampicillin (100 mg/L) at 37°C and 200rpm. Cells were then inoculated into liquid LB medium and cultured to OD_600_ = 0.6–0.8 (Park et al., 2023). Production of recombinant F4′OMT (PaF4′OMT, MpOMT4, SOMT2, CreOMT1, CreOMT4) or 1,6RhaT (Cs1,6RhaT, CiRhaT-GD4x, CiRhaT-AH2x) proteins fused to glutathione S-transferase (GST) was induced using 100 μM isopropyl β-D-1-thiogalactopyranoside (IPTG) at 20°C for 3 h. Small aliquots of the induced cultures were set aside for SDS-PAGE analysis. Diosmetin or diosmetin7-*O*-β-D-glucopyranoside (diosmetin7-*O*-glucoside) (100 μM) was added as a substrate depending on the experiment. After 3 h of incubation at 28°C, the culture medium was extracted with an equal volume of ethyl acetate. After centrifugation at 13,000 rpm for 10 min at 4°C (MDX-310, Tomy Seiko Co., Ltd., Japan), the upper ethyl acetate phase was transferred to a fresh tube and evaporated under N_2_ gas. The residue was dissolved in 80% (v/v) methanol and analyzed by high-performance liquid chromatography (HPLC), ultra-performance liquid chromatography (UPLC), and UPLC-DAD-QToF/MS.

### Transient expression of diosmin biosynthetic genes in *Nicotiana benthamiana* leaves

Agrobacterium strain GV3101 harboring the overexpression vectors was transformed using the freeze-thaw method (Weigel and Glazebrook, 2006). A positive colony of GV3101 transformant was picked and pre-cultured in YEP medium with antibiotics at 28°C for 24 h. For the main culture, 50 μl of pre-culture was transferred into a fresh 50 ml of YEP medium containing antibiotics. This cell culture was centrifuged at 5,000 rpm for 10 min (MDX-310, Tomy Seiko Co., Ltd., Japan). The pellet was resuspended in agro-infiltration buffer containing 10 mM MES (pH 5.6), 10 mM MgCl_2_, 5% (w/v) D-glucose, and 200 µM acetosyringone (4-hydroxy-3,5-dimethoxyacetophenone, Sigma) to a final OD_600_ = 0.6. Equal volumes of Agrobacterium cells (OD_600_ = 0.6) carrying the overexpression vector and those containing a construct harboring *P19*, which is a suppressor of RNA-mediated gene silencing, were mixed and incubated at room temperature for 2–3 h. *N. benthamiana* plants were grown in a greenhouse with a 16-h/8-h light/dark cycle (at 28°C during the day and 25°C at night). Agrobacterium cells in infiltration buffer were mixed thoroughly before co-infiltration. Agrobacterium mixtures were gently infiltrated using a 1-ml blunt-end syringe against the abaxial side of the leaves of 4-week-old *N. benthamiana* plants. Three individual plants were infiltrated with each construct or GV3101 (GV) cells used as a control. After agro-infiltration, the plants were grown in the same greenhouse for a further six days, and infiltrated leaves were then frozen in liquid nitrogen and stored at −80°C until use.

### Identification of diosmetin by HPLC/UPLC analysis

Flavonoid contents of agro-infiltrated *N. benthamiana* leaves were quantified as their aglycone forms generated by acid hydrolysis. Ground leaf tissue (100 mg FW) was mixed with 400 μl 80% (v/v) methanol and incubated overnight at 4°C. After centrifugation at 13,000 rpm for 10 min at 4°C, 200 μl of the supernatant was transferred to a new tube and was subjected to acid hydrolysis by adding 600 μl 1 M HCl and incubation at 94°C for 2 h. Flavonoids were then extracted using 800 μl of ethyl acetate, and the upper ethyl acetate phase was evaporated under N_2_ gas. The residue was dissolved in 80% (v/v) methanol, filtered through a 0.2-μm Teflon polytetrafluoroethylene (PTFE) hydrophilic syringe filter (Thermo Fisher Scientific, USA), and subjected to analysis performed on a HPLC (High Performance Liquid Chromatography) system (Shimadzu, Japan) with the method described in Park et al. (2021) and an LC-20A UPLC (Ultra Performance Liquid Chromatography) system (Shimadzu) equipped with an Inertsil-ODS3 C18 column (3 μm, 3.0 × 100 mm; Shimadzu). For UPLC analysis, the mobile phase consisted of 0.1% (v/v) formic acid, acetonitrile, and methanol (60:16:24, v/v/v) at a constant flow rate of 0.3 ml/min (Chen et al., 2012). The temperature of the column was maintained at 30°C. A diode array detector was used for real-time monitoring of the chromatograms, and the spectra of the compounds were recorded between 210 nm and 800 nm. Flavonoids, including flavonol (quercetin), flavones (luteolin, apigenin), and methylated flavones (chrysoeriol, diosmetin), were analyzed at 350 nm. The compounds were identified by comparing their retention times and UV spectra to those of flavonoid aglycone standards analyzed by HPLC or UPLC.

### Identification of flavonoid aglycones and glycosides using UPLC-DAD-QToF/MS

The ground powder (100 mg FW) of agro-infiltrated *N. benthamiana* leaves was incubated overnight with 80% (v/v) methanol at 4°C. After centrifugation at 13,000 rpm for 10 min at 4°C, half of the extract was used for acid hydrolysis of flavonoids, and the other half was used for analysis of flavonoid glycosides. The extracts were injected into an ultra-performance liquid chromatography diode array detector (UPLC-DAD) system (SCIEX Co., USA) connected to a reverse-phase column (CORTECS UPLC T3, 2.1 × 150 mm I.D., 1.6 μm; Waters Co., Milford, MA, USA) and a CORTECS UPLC VanGuard™ T3 pre-column (2.1 × 50 mm I.D., 1.6 μm; Waters Co.). The mobile phase consisted of water containing 0.5% (v/v) formic acid (Sol. A) and acetonitrile containing 0.5% (v/v) formic acid (Sol. B). A gradient program was used, with the following mobile phase compositions at the times indicated (in v/v): 20 min, 25% B; 25 min, 50% B; 30 min, 90% B; 32 min, 90% B; 35 min, 5% B; 40 min, 5% B, with a flow rate of 0.3 ml/min. Flavonoid aglycones and glycosides were characterized using a quadrupole time-of-flight mass spectrometer (QToF-MS) (SCIEX Co.) in positive or negative ionization mode. Mass spectrometry conditions were maintained as follows: ion source gas, 50 psi; curtain gas, 30 psi; ion source temperature, 450°C; declustering potential (DP), 80 V; collision energy (CE), 15 ± 10 V; spray voltage, 5500 V; scan ranges of *m*/*z* 100–1200.

### Chemical standards

Quercetin, naringenin, eriodictyol, apigenin, luteolin, and diosmetin were purchased from Sigma-Aldrich (USA). Diosmetin 7-*O*-β-D-glucopyranoside (diosmetin 7-*O*-glucoside) was purchased from MedChemExpress (USA), and diosmin was purchased from Supelco (USA). The flavonoids were prepared as 100 mM stock solution in DMSO.

## Results

### Optimization of luteolin production in *N benthamiana*

**Figure 2:**
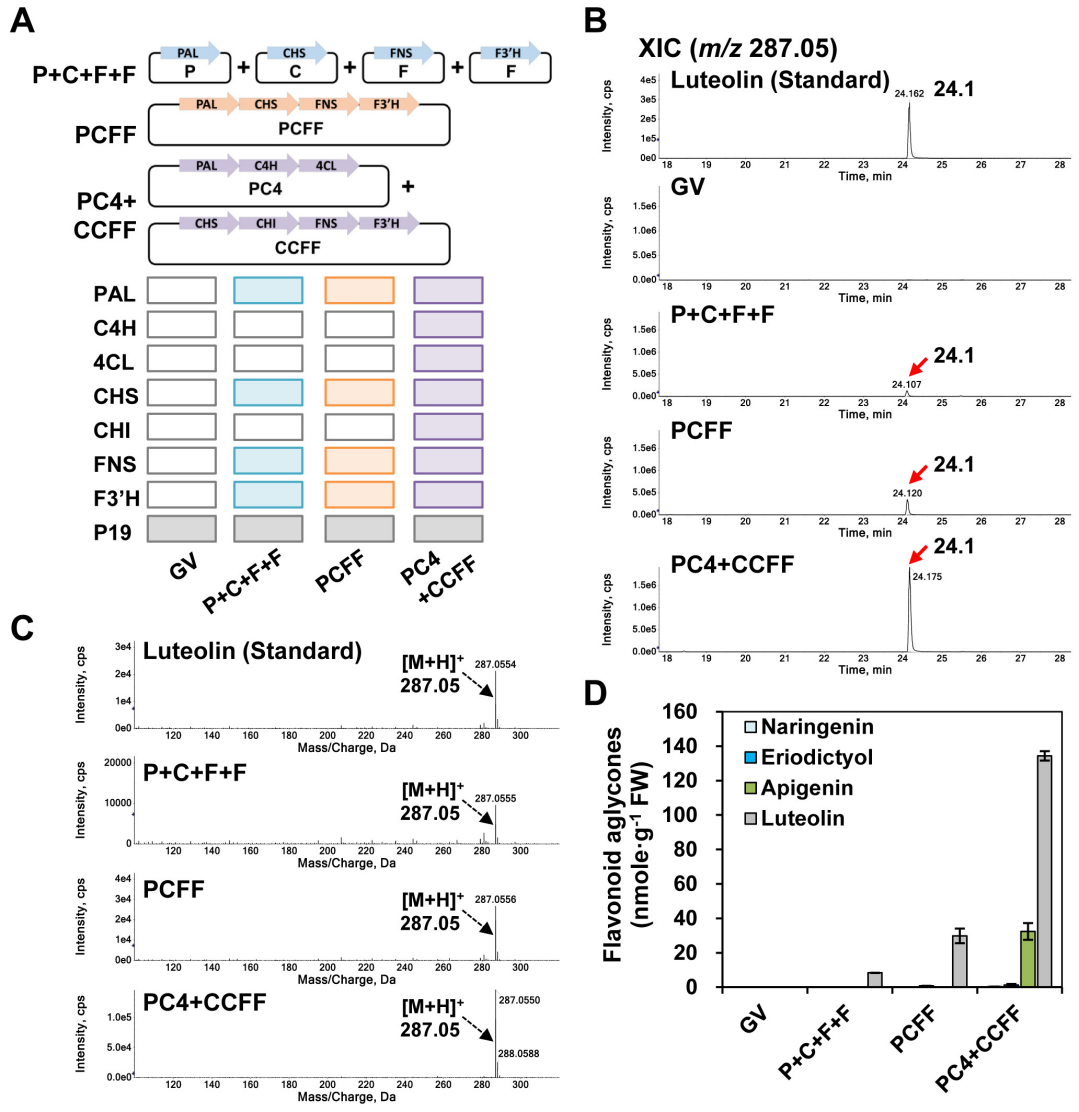
Luteolin production in *N. benthamiana* leaves with differently reconstituted flavonoid biosynthesis modules. *N. benthamiana* leaves were agro-infiltrated with *Agrobacterium tumefaciens* strain GV3101 harboring the single gene expression cassettes P+C+F+F (P, AtPAL; C, OsCHS; F, OsFNS; F, OsF3′H) or the multigene expression cassettes PCFF (P, AtPAL; C, OsCHS; F, OsFNS; F, OsF3′H), or PC4 (P, AtPAL; C, AtC4H; 4, Sh4CL) and CCFF (C, OsCHS; C, BrCHI; F, OsFNS; F, OsF3′H) in binary vectors. GV3101 (GV) was used as a negative control. UPLC-DAD-QToF/MS analysis of extracts from leaves was conducted in positive ion mode. **(A)** Schematic diagram of single (P+C+F+F) or multigene (PCFF, PC4, CCFF) expression vectors of target genes. **(B)** Extracted-ion chromatogram (XIC) at *m/z* 287.05 representing protonated luteolin aglycone in infiltrated leaves. **(C)** Representative XICs showing mass spectra of the luteolin standard and products detected in infiltrated leaves. **(D)** Flavonoid contents in the three independent biological samples were calculated based on the area of standards (naringenin, eriodictyol, apigenin, luteolin). The mean values ± SE (standard error) of three independent biological samples are shown.

The plant phenylpropanoid and flavonoid biosynthetic pathways from phenylalanine to luteolin are well characterized (Ferrer et al., 2008; Muruganathan et al., 2022) (**Figure 1**). Thus, to reconstitute the biosynthetic pathway that would yield the highest amounts of luteolin, we designed, tested, and compared three different gene expression systems (P+C+F+F, PCFF, PC4+CCFF). PAL and CHS are the rate-limiting enzymes in the phenylpropanoid pathway and flavonoid pathway, respectively, and FNS and F3′H catalyze C2-C3 double bond formation and B-ring hydroxylation of flavonoids, respectively. Therefore, we chose the four key genes *PAL* (P), *CHS* (C), *FNS* (F), and *F3′H* (F) and all seven genes [*PAL* (P), *C4H* (C), *4CL* (4), *CHS* (C), *CHI* (C), *FNS* (F), F*3′H* (F)] in the flavonoid biosynthesis pathway to produce luteolin. We constructed three types of modules, namely PCFF (PAL, CHS, FNS, F3′H), PC4 (PAL, C4H, 4CL), or CCFF (CHS, CHI, FNS, F3′H) in each vector. Then, we co-infiltrated the vectors harboring the individual genes (P+C+F+F) or infiltrated vectors containing multigene expression modules (PCFF or PC4+CCFF) in *N. benthamiana* leaves (**Figure 2A**).

We analyzed the infiltrated leaves by UPLC-DAD-QToF/MS in positive ionization mode to compare the luteolin productivity of each module (**Figures 2B–D**). We detected and quantified luteolin through extracted-ion chromatograms (XICs) and mass profiles. In the QToF/MS analysis, luteolin (*m/z* 287.05 [M+H]^+^) had a retention time of 24.1 min in agro-infiltrated leaves for all three combinations of expression constructs (P+C+F+F; PCFF; PC4+CCFF) (**Figures 2B, C**). Luteolin content was 3.6-fold higher in leaves infiltrated with the PCFF (29.8 nmol/g FW) module than in those co-infiltrated with the individual constructs (P+C+F+F [8.4 nmol/g FW]). Furthermore, luteolin content was 4.5-fold higher in leaves co-infiltrated with the PC4+CCFF (134.4 nmol/g FW) modules than in those infiltrated with the PCFF module alone. Apigenin (32.3 nmol/g FW) was only detected in leaves co-infiltrated with the PC4+CCFF modules (**Figure 2D**). Thus, infiltration with the multigene expression modules resulted in higher levels of luteolin than did expression of the individual genes, and the PC4+CCFF modules together yielded higher levels of luteolin than did the PCFF module, indicating that overexpression of all seven genes in the flavonoid biosynthesis pathway resulted in the greatest accumulation of luteolin.

### Selection of an optimal F4′OMT to produce diosmetin from luteolin

**Figure 3:**
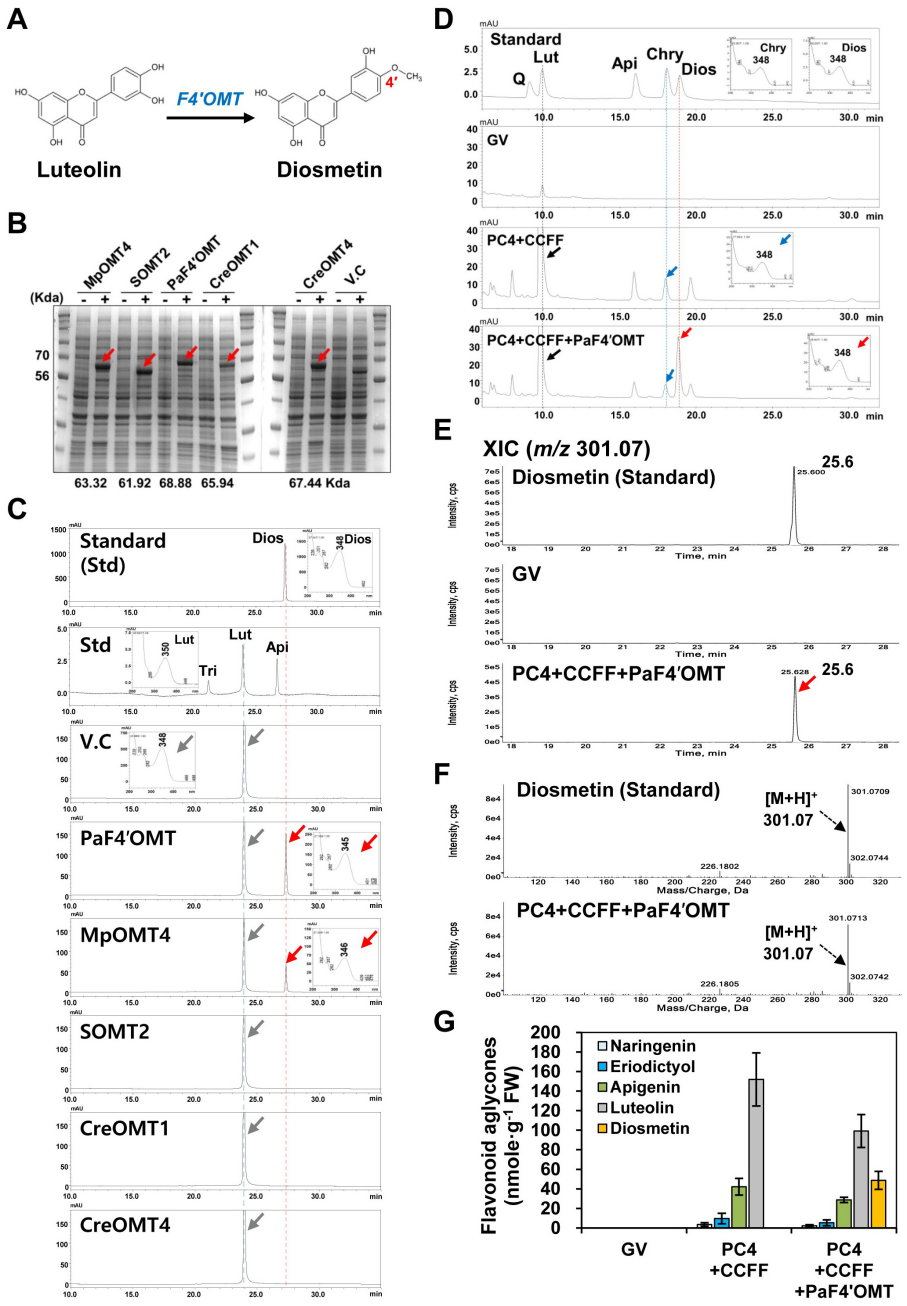
Screening of an optimum F4′OMT for diosmetin production. **(A)** Diosmetin is produced from luteolin by flavonoid-4′-*O*-methyltransferase (F4′OMT). **(B)** Expression of recombinant F4′OMT enzymes in *E*. *coli* Rosetta (DE3). Proteins were separated on a 10% SDS-polyacrylamide gel. V.C, vector control. **(C)** Substrate-feeding assays of recombinant flavonoid 4′-*O*-methyltransferases (F4′OMTs) using luteolin as the substrate. PaF4′OMT (*Plagiochasma appendiculatum*); MpOMT (*Mentha* × *piperita*); SOMT2 (*Glycine max*); CreOMT1 (*Citrus reticulata*); CreOMT4 (*C. reticulata*). **(D)** UPLC analysis of diosmetin standard and flavonoid extracts from leaves infiltrated with mixtures of *Agrobacterium* GV3101 strains harboring binary vectors containing multigene expression cassettes [PC4+CCFF: PC4 (P, AtPAL; C, AtC4H; 4, Sh4CL); CCFF (C, OsCHS; C, BrCHI; F, OsFNS; F, OsF3′H)] and a single (PaF4′OMT) gene expression cassette. GV3101 (GV) was used as a negative control. **(E)** Flavonoid aglycone profiles in the infiltrated leaves were analyzed by UPLC-DAD-QToF/MS in positive ion mode. Extracted-ion chromatogram (XIC) at *m/z* 301.07 of protonated diosmetin aglycone in the infiltrated leaves. **(F)** Representative XICs showing mass spectra of diosmetin standard and products in the infiltrated leaves. **(G)** Flavonoid contents in three independent biological samples were calculated based on the area of corresponding standards (naringenin, eriodictyol, apigenin, luteolin, and diosmetin). Mean values ± SE (standard error) of three independent biological samples are shown.

4′-*O*-methylation of luteolin to generate diosmetin is catalyzed by F′4OMT (**Figures 1**, **3A**). To select the optimal *F4′OMT* gene, we compared the enzyme activities of five candidate F4′OMTs (PaF4′OMT, MpOMT4, SOMT2, CreOMT1, CreOMT2) previously reported (Kim et al., 2005; Liu et al., 2017; Zohra et al., 2020; Liu et al., 2022). To examine the efficiency of diosmetin production, we conducted a substrate-feeding assay with luteolin as the substrate. Successful production of the recombinant proteins following induction by IPTG was demonstrated by SDS-PAGE analysis (**Figure 3B**). Bacterial cultures expressing these *F4′OMT*s fed with luteolin were extracted and analyzed by HPLC, which revealed that PaF4*′OMT* or *MpOMT4* produced diosmetin (RT: 27.3 min), whereas those three OMTs (*SOMT2*, *CreOMT1*, *CreOMT4*) did not. As PaF4′OMT produced the most diosmetin from luteolin (**Figure 3C**), we decided to co-express *PaF4′OMT* with the PC4+CCFF modules in *N. benthamiana.*

We infiltrated *N. benthamiana* leaves with the PC4+CCFF or PC4+CCFF+PaF4′OMT constructs, and analyzed their acid-hydrolyzed extracts by UPLC. Luteolin produced by expression of the PC4+CCFF modules was methylated to chrysoeriol, but diosmetin was newly synthesized in the leaves co-infiltrated with PC4+CCFF+PaF4′OMT, indicating that PaF4′OMT successfully catalyzed the 4′*-O-*methylation of luteolin (**Figure 3D**). QToF/MS analysis of the leaves co-infiltrated with the PC4+CCFF+PaF4′OMT constructs identified diosmetin (*m/z* 301.07 [M+H]^+^) at a retention time of 25.6 min (**Figures 3E, F**). Diosmetin was produced only in the PC4+CCFF+PaF4′OMT combination (48.7 nmol/g FW) as much as luteolin content decreased (52.7 nmol/g FW) (**Figure 3G**). These results indicate that luteolin is successfully converted to diosmetin by *PaF4′OMT* expression in agro-infiltrated leaves but two-thirds of the luteolin (99.2 nmol/gFW) is still present without methylation.

### 1 Examination of glucosyltransferase CsUGT76Factivity for diosmetin 7-*O*-glucoside production in *N. benthamiana*

**Figure 4:**
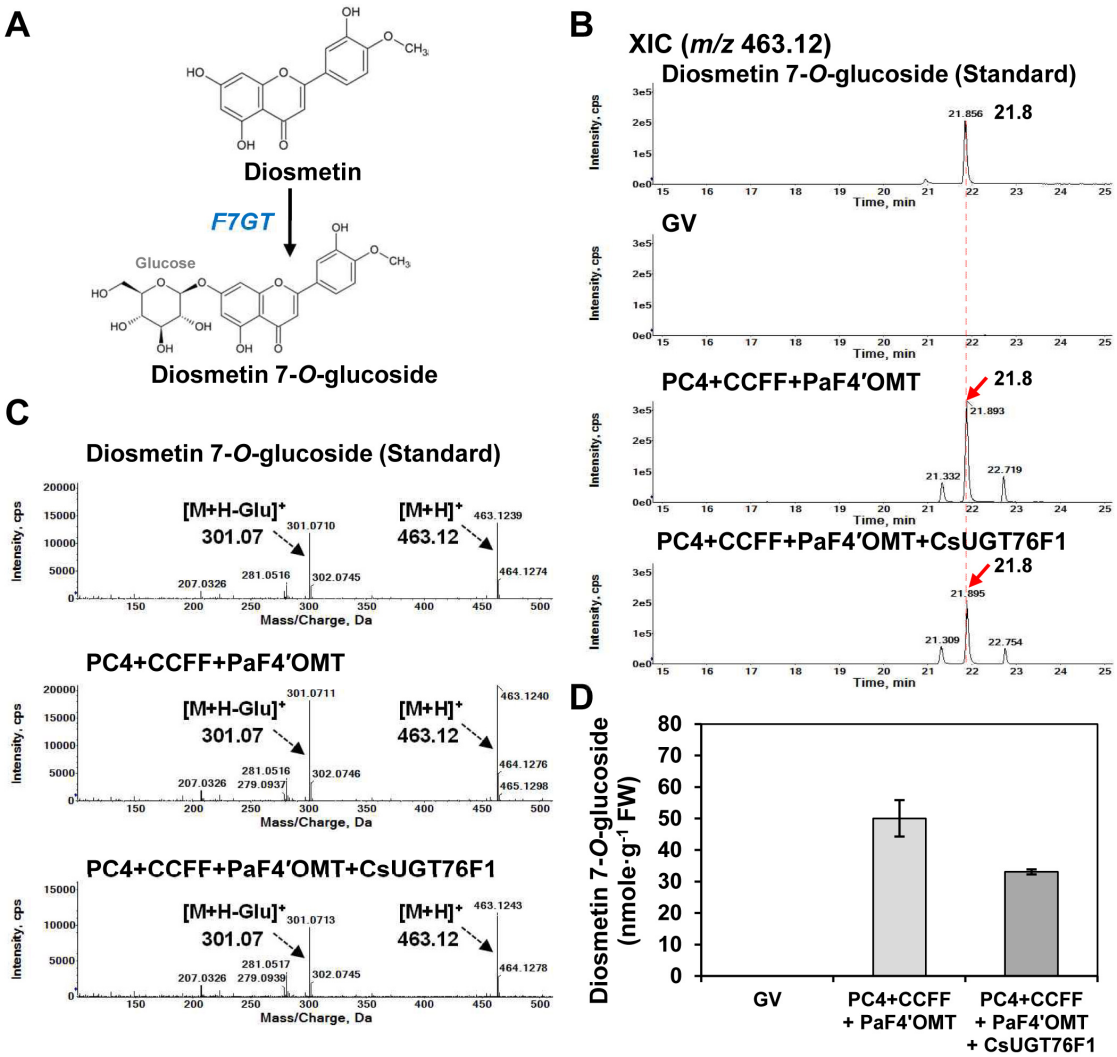
Impact of co-expressing glucosyltransferase with the diosmetin production module in *N. benthamiana. N. benthamiana* leaves were agro-infiltrated with *Agrobacterium tumefaciens* GV3101 strains harboring binary vectors containing multigene expression cassettes [PC4+CCFF: PC4 (P, AtPAL; C, AtC4H; 4, Sh4CL); CCFF (C, OsCHS; C, BrCHI; F, OsFNS; F OsF3′H)] and single gene expression cassettes [PaF4′OMT, CsUGT76F1]. Flavonoid profiles in the infiltrated leaves were analyzed by UPLC-DAD-QToF/MS in positive ion mode. **(A)** Diosmetin 7-*O*-glucoside is produced from diosmetin by flavonoid 7-*O*-glucosyltransferase (F7GT). **(B)** XICs at *m/z* 463.12 representing protonated diosmetin-7-*O*-glucoside in *N. benthamiana* leaves agro-infiltrated with a GV3101 strain harboring binary vectors containing multiple genes (PC4+CCFF, CCFF) and single gene (PaF4′OMT, F7GT). **(C)** Representative XICs showing mass spectra of diosmetin-7-*O*-glucoside standard and products in the infiltrated leaves. **(D)** Diosmetin-7-*O*-glucoside contents in three independent biological samples were calculated based on the area of the standards. Mean values ± SE (standard error) of three independent biological samples are shown.

Flavonoid 7-*O*-glucosyltransferase (F7GT) is well-characterized in *Citrus* species, and diosmetin 7-*O*-glucoside is reported to be produced by CsUGT76F1 *in vitro* (Liu et al., 2018) (**Figure 4A**).

Thus, we transiently co-expressed *CsUGT76F1* with the PC4+CCFF+PaF4′OMT constructs in *N. benthamiana* leaves and assessed the ability of this combination of constructs to produce diosmetin 7-*O*-glucoside. The infiltrated leaves were extracted with 80% methanol and analyzed by UPLC-DAD-QToF/MS in positive ionization mode. Diosmetin 7-*O*-glucoside (*m/z* 463.12 [M+H]^+^) was detected in leaves co-infiltrated with PC4+CCFF+PaF4′OMT (50.1 nmol/g FW) or PC4+CCFF+PaF4′OMT+CsUGT76F1 (33 nmol/g FW) (**Figure 4B**). Unexpectedly, diosmetin 7-*O*-glucoside production was lower in leaves co-infiltrated with PC4+CCFF+PaF4′OMT+CsUGT76F1 than in those co-infiltrated with PC4+CCFF+PaF4′OMT (**Figures 4B, D**). These results indicate that the 7-*O*-glycosylation of diosmetin can be carried out effectively by endogenous F7GT activity in *N. benthamiana.* Even though these results suggest that *CsUGT76F1* expression is unnecessary to catalyze the production of diosmetin 7-*O*-glucoside in *N. benthamiana*, we decided to include *CsUGT76F1* in the set of constructs used for diosmin biosynthesis, so that our expression system could be used in other hosts with different levels of endogenous F7GT activity.

### Selection of an 1,6RhaT for diosmin production

**Figure 5:**
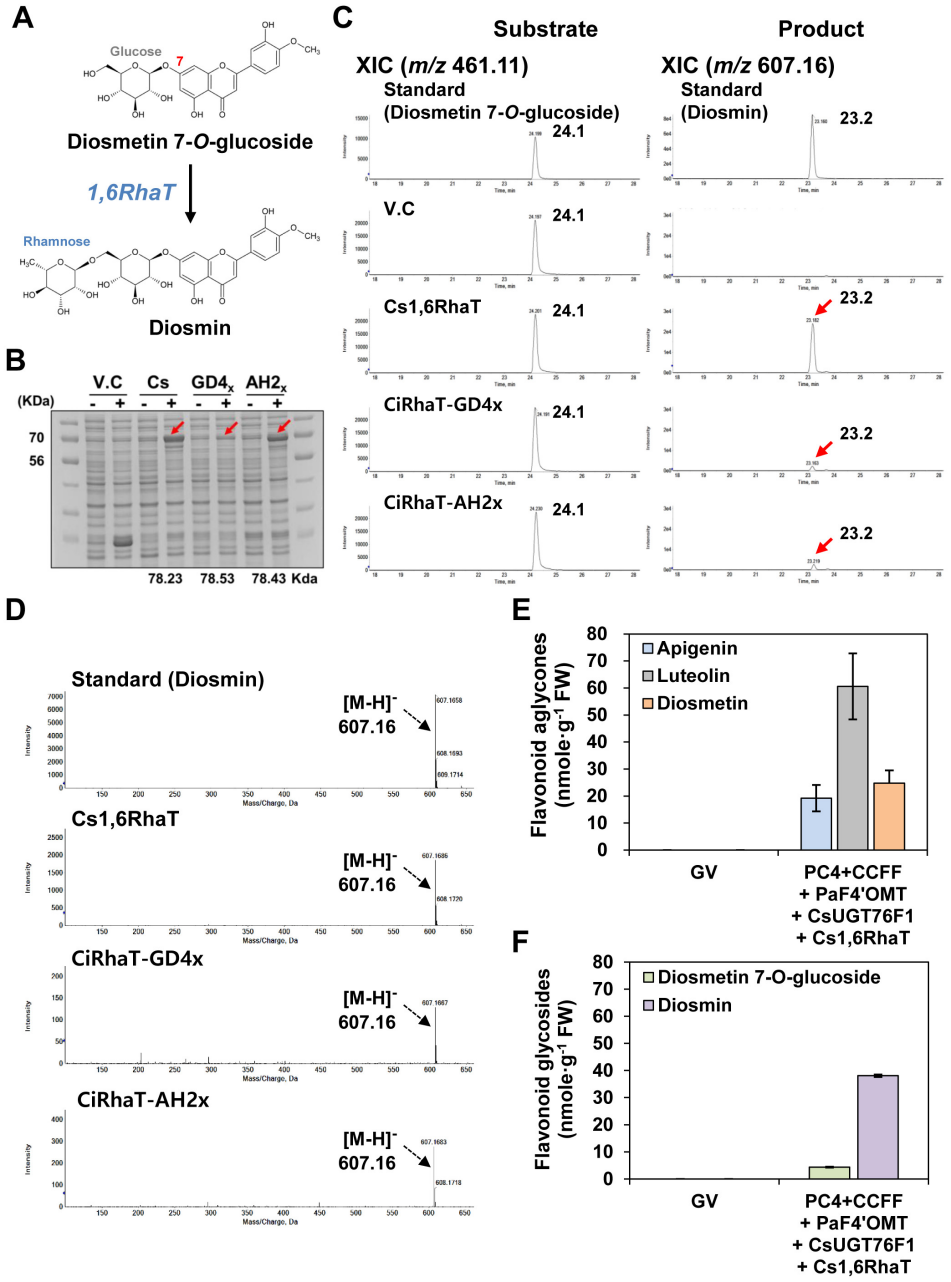
Diosmin production in *N. benthamiana* leaves by multigene expression and co-expression of target genes. Flavonoid profiles in *E*. *coli* were analyzed by UPLC-DAD-QToF/MS in negative ion mode. **(A)** Diosmin is produced by 1,6-rhamnosyltransferase (1,6RhaT) from diosmetin 7-*O*-glucoside. Substrate-feeding assays of recombinant 1,6-rhamnosyltranferase (1,6RhaT). **(B)** Proteins were separated on a 10% SDS-polyacrylamide gel. V.C, vector control. Cs1,6RhaT (*Citrus sinensis*), CiRhaT-GD4x (GD4x, *Chrysanthemum indicum*), CiRhaT-AH2x/CiRhaT-HB2x (AH2x, *Chrysanthemum indicum*). **(C)** Extracted-ion chromatograms (XICs) at *m/z* 461.11 and *m/z* 607.16 representing deprotonated diosmetin 7-*O*-glucoside and diosmin in *E*. *coli* Rosetta (DE3). **(D)** Representative XICs showing mass spectra of diosmin standard and products extracted from *E*. *coli* expressing recombinant RhaT enzymes. **(E)** Flavonoid aglycones (apigenin, luteolin, diosmetin) contents *in N. benthamiana* leaves agro-infiltrated with GV3101 strains harboring binary vectors containing multiple genes [PC4+CCFF: PC4 (P, AtPAL; C, AtC4H; 4, Sh4CL); CCFF (C, OsCHS; C, BrCHI; F, OsFNS; F, OsF3′H)] and single genes [PaF4′OMT, CsUGT76F1, Cs1,6RhaT]. **(F)** Flavonoid glycosides (diosmetion-7-*O*-glucoside and diosmin) contents *in N. benthamiana* leaves agro-infiltrated with GV3101 strains harboring binary vectors containing multiple genes (PC4+CCFF) and single genes (PaF4′OMT, CsUGT76F1, Cs1,6RhaT). Average flavonoid aglycone and glycoside contents were calculated based on the areas of the corresponding standards. Mean values ± SE (standard error) of three independent biological samples are shown.

Diosmin is biosynthesized from diosmetin 7-*O*-glucoside in a reaction catalyzed by 1,6-rhamnosyltransferase (1,6 RhaT) (**Figure 5A**). We examined the ability of three previously reported *1,6RhaT* genes, i.e., *Cs1,6RhaT*, *CiRhaT-GD4x*, and *CiRhaT-AH2x* (Frydman et al., 2013; Wu et al., 2022), to produce diosmin when heterologously expressed in *N. benthamiana*. To this end, we conducted a substrate-feeding assay with the enzymes encoded by these three genes and diosmetin 7-*O*-glucoside as a substrate. SDS-PAGE analysis revealed that two recombinant RhaT proteins (Cs1,6RhaT and CiRhaT-AH2x) were produced upon IPTG induction, whereas recombinant CiRhaT-GD4x was poorly induced (**Figure 5B**).

The bacterial cultures expressing *1,6RhaT*s supplied with diosmetin 7-*O*-glucoside were extracted and analyzed by UPLC-DAD-QToF/MS in negative ionization mode to detect diosmin formation. QToF/MS analysis showed that the cells expressing *Cs1,6RhaT* produced the most diosmin (*m/z* 607.16 [M-H]^−^) (**Figures 5C, D**). To produce diosmin in *N. benthamiana*, *Cs1,6RhaT* was transiently co-infiltrated with the PC4+CCFF+PaF4′OMT+CsUGT76F1 constructs (**Figures 5E, F**). QToF/MS analysis showed that the leaves co-infiltrated with the ten selected genes (i.e., PC4+CCFF+PaF4′OMT+CsUGT76F1+Cs1,6RhaT) successfully produced diosmin (38.1 nmol/g FW) (**Figure 5F**). By determining the enzyme catalyzing the final stage, the 6′′*-O-*rhamnosylation of diosmetin 7*-O-*glucoside, we completed the selection of 10 genes (from *PAL* to *RhaT*) to reconstitute the diosmin biosynthesis pathway.

### Production of diosmin by reconstitution of the diosmin biosynthetic pathway

**Figure 6:**
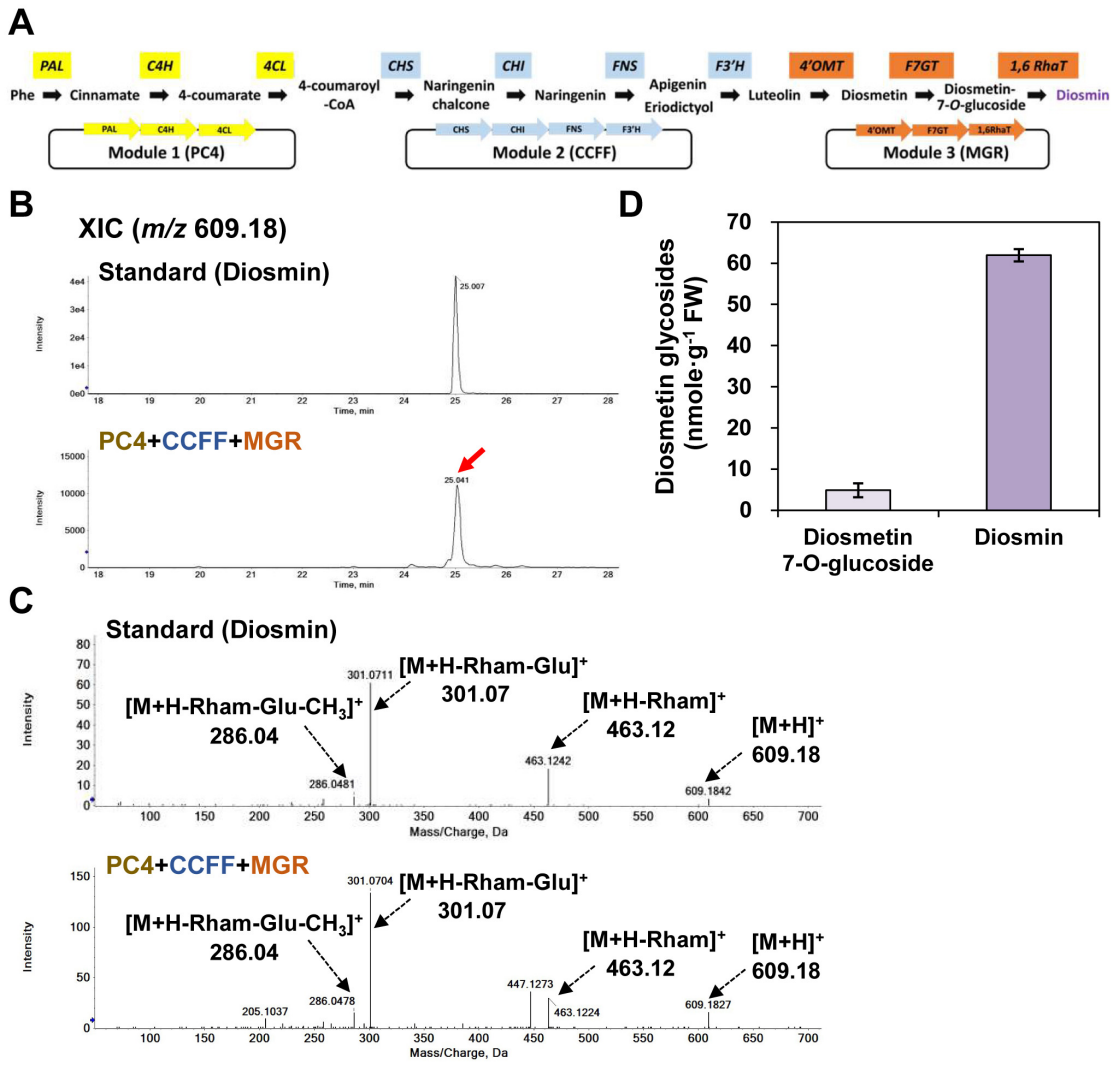
Diosmin production in *N. benthamiana* by transient co-expression of three modules reconstituting the diosmin biosynthesis pathway. *N. benthamiana* leaves were agro-infiltrated with GV3101 strains harboring binary vectors containing three modules: PC4 (P, AtPAL; C, AtC4H; 4, Sh4CL), CCFF (C, OsCHS; C, BrCHI; F, OsF3′H; F, OsFNS), and MGR (M, PaF4′OMT; G, CsUGT76F1; R, Cs1,6RhaT). Flavonoid glycoside profiles in the infiltrated leaves were analyzed by UPLC-DAD-QToF/MS in positive ion mode. **(A)** Schematic diagram of multigene expression vectors for target genes; module 1, PC4 (P, AtPAL; C, AtC4H; 4, Sh4CL); module 2, CCFF (C, OsCHS; C, BrCHI; F, OsF3′H; F, OsFNS); module 3, MGR (M, PaF4′OMT; G, CsUGT76F1; R, Cs1,6RhaT). **(B)** Extracted-ion chromatogram (XIC) at *m/z* 609.18 representing protonated diosmin standard and products from the infiltrated leaves. **(C)** Representative XICs at *m/z* 609.18 showing MS/MS spectra of diosmin standard and products from the infiltrated leaves. **(D)** Flavonoids glycosides (diosmetin 7-*O*-glucoside, diosmin) contents in the agro-infiltrated leaves. Average diosmetin 7-*O*-glucoside and diosmin contents were calculated based on the areas of the corresponding standards. Mean values ± SE (standard error) of three independent biological samples are shown.

For optimum production of diosmin in *N. benthamiana* leaves, we constructed three modules [module 1, PC4; module 2, CCFF; module 3, MGR (PaF4′OMT+CsUGT76F1+Cs1,6RhaT)] that together contained the ten genes selected above (**Figure 6A**). We transiently co-infiltrated the three modules (PC4+CCFF+MGR) into *N. benthamiana* leaves and evaluated the capacity of these leaves to produce diosmin (**Figures 6B–D**). UPLC-DAD-QToF/MS analysis in positive ionization mode detected a protonated ion at *m/z* at 609.18 [M+H]^+^ corresponding to that of diosmin in leaves co-infiltrated with the PC4+CCFF+MGR modules (**Figures 6B, C**). The MS/MS spectra showed a protonated molecular ion (*m/z* 609.18 [M+H]^+^) and fragment ions at *m/z* 463.12 ([M+H-rham]^+^), *m/z* 301.07 ([M+H-rham-Glu]^+^), and *m/z* 286.04 ([M+H-rham-Glu-CH_3_]^+^), suggesting the loss of a rhamnose; a rhamnose and a glucose; and a rhamnose, a glucose, and a methyl group, respectively, and these corresponded to the fragment ions of the diosmin standard. Thus, we identified diosmin based on its fragmentation pattern (**Figure 6C**). By co-infiltrating the PC4+CCFF+MGR modules in *N. benthamiana* leaves, a substantial amount of diosmin was produced (61.9 nmol/g FW), while diosmetin 7-*O*-glucoside was barely detected (4.8 nmol/g FW) as it was mostly converted to diosmin (**Figure 6D**). The diosmin content of *N. benthamiana* leaves co-expressing the three modules (PC4+CCFF+MGR) was 1.7-fold higher than that of leaves co-expressing two modules and the genes encoding the enzymes that catalyze the last three steps of the pathway individually (PC4+CCFF+PaF4′OMT+CsUGT76F1+Cs1,6RhaT) (**Figures 5D**, **6D**). Thus, diosmin production is more efficient when all the genes in the pathway are grouped into modules than when some of them are expressed individually.

Other flavone dihexosides accumulated in leaves co-infiltrated with the PC4+CCFF+MGR modules. XIC and MS/MS spectra suggested these dihexosides were apigenin 7-*O*-rutionoside (12.2 nmol/g FW) and luteolin 7-*O*-rutinoside (33.4 nmol/g FW), respectively (**Supplementary Figure 1**), and accounted for approximately 18% and 50% of the diosmetin glycoside contents (**Figure 6D**). Thus, F3′H and F4′OMT activities are not sufficient to convert most luteolin to diosmin in this system. However, the glucosyltransferase and rhamnosyltransferase activities appeared to completely cover the metabolic flow, since all detected flavones underwent rutinosylation. These results show that we successfully constructed a three-module expression system that can be used to produce diosmin in heterologous plants.

## Discussion

Several recent studies on metabolic engineering have focused on reconstituting entire biosynthetic pathways, including upstream metabolites, using multigene expression systems to enhance the production of target metabolites (Ogo et al., 2013; Kim et al., 2014; Wang et al., 2021b). Kim et al. (2014) reported that overexpression of *SbC4H* and *Sb4CL* in *Scutellaria baicalensis* hairy root lines increased the total flavone content approximately threefold and produced flavones such as baicalin, baicalein, and wogonin at concentrations of approximately 5–62 mg/g DW. Vinblastine precursors, including precondylocarpine acetate, have been produced by multigene expression in *N. benthamiana*, at concentrations of up to 2.7 mg/g FW (Grzech et al., 2022). Similarly, the anticancer drug paclitaxel was produced in *Nicotiana benthamiana* through reconstitution of its early biosynthetic pathway comprising six enzymes (Liu et al., 2024). We reasoned that the successful production of luteolin, an upstream metabolite of diosmin, was necessary for the production of high concentrations of diosmin. We tried to direct metabolic flux from phenylalanine to diosmin by reconstituting the entire biosynthetic pathway. First, we used the PC4 module, expressing *PAL*, *C4H*, and *4CL*, to catalyze the reactions in the phenylpropanoid pathway. Then, we constructed the CCFF module, consisting of *CHS*, *CHI*, *FNS*, and *F3′H*, to catalyze the reactions in the flavone biosynthetic pathway and produce luteolin. Co-expression of the PC4+CCFF modules yielded amounts of luteolin (134.4 nmol/g FW) that were 4.5-fold higher than those obtained from PCFF expression alone. In a previous study, it was shown that the expression of all eight genes from *PAL* to *cinnamyl alcohol dehydrogenase* (*CAD*) of the coniferyl alcohol pathway increased the yield of (–)-deoxypodophyllotoxin (DPT) approximately 10-fold (Schultz et al., 2019). Thus, it appears that the phenylpropanoid pathway must be activated to produce substantial amounts of flavonoids and their derivatives.

Flavonoid profiling of *N. benthamiana* leaves overexpressing PC4+CCFF showed that eriodictyol was barely detected, while one-third of luteolin was converted to apigenin. This finding suggests that OsFNS activity is sufficient to convert naringenin to apigenin and eriodictyol to luteolin (Lam et al., 2014). We also suggest that the OsF3′H cannot completely convert apigenin into luteolin (**Figure 2D**).

Only 30% of all luteolin produced in tobacco leaves was converted to diosmetin (**Figure 3G**). Considering the study of Liu et al. (2017), the modest level of PaF4′OMT activity might be because this enzyme has a lower substrate preference for luteolin than for apigenin. Another possibility is that an endogenous 7-*O*-glucosyltransferase converts most of the luteolin into luteolin glycoside and that PaF4′OMT cannot methylate the luteolin glucoside, in agreement findings reported by Liu et al. (2020). Small amounts of chrysoeriol were detected in *N. benthamiana* leaves co-expressing PC4+CCFF, suggesting that endogenous 3′*-O-*methyltransferase activity is present in *N. benthamiana*. However, chrysoeriol contents were slightly lower in leaves co-expressing PC4+CCFF+PaF4′OMT. We suspect that overexpressing *PaF4′OMT* overwhelmed the endogenous 3′-*O*-methyltransferase activity and that PaF4′OMT exhibits high substrate specificity.

In this study, we chose Cs1,6RhaT as the rhamnosyltransferase with the highest activity for diosmin production. We demonstrated that leaves transiently co-expressing *Cs1,6RhaT* and PC4+CCFF+PaF4′OMT+CsUGT76F1 produced diosmin. Around 87% of all diosmetin 7-*O*-glucoside was converted to diosmin (**Figures 4D**, **5F**). Rhamnosylation was not achieved by the endogenous rhamnosyltransferase in *N. benthamiana*. Therefore, heterologous Cs1,6RhaT was used to catalyze the final step of diosmin production. Since modularization is more efficient to produce end products than introducing individual genes, we constructed the MGR (PaF4′OMT+CsUGT76F1+Cs1,6RhaT) module and used it to generate a three-module system (PC4+CCFF+MGR). The three compressed PC4+CCFF+MGR modules produced significantly higher amounts of diosmin than the PC4+CCFF modules co-expressed with *PaF4′OMT*, *CsUGT76F1*, and *Cs1,6RhaT* individually (**Figure 6**).

Natural plant products have been produced in microbial hosts such as *E. coli* and *Saccharomyces cerevisiae* through reconstitution of the corresponding biosynthetic pathways (Yu et al., 2011). A few studies have described the production of flavonoids such as baicalein and scutellarein by expression of multiple biosynthetic genes (*PrPAL*, *Pc4CL*, *PhCHS*, *MsCHI*, *PcFNS1/SbFNSII*, and *SbF6H*) in genetically engineered *E. coli*, but various supplements, such as glucose, NH_4_Cl, and phenylalanine or tyrosine were required in these systems (Li et al., 2019). Plant systems are more suitable for producing plant-derived products such as valuable pharmaceutical agents because they can compartmentalize metabolites within their cells and specific tissues and do not require any materials except light, water, and mineral nutrients.

Through transient expression of the PC4+CCFF+MGR three-module system comprising all genes of the diosmin biosynthesis pathway, from *PAL* to *1,6-rhamnosyltransferase*, it was possible to produce substantial amounts of diosmin (37.7 ± 0.9 µg/g FW) with stable expression of the target genes in *N. benthamiana* (**Supplementary Figure 2**). This is the first report, to our knowledge, of diosmin production in a heterologous plant system without having to provide substrates. The successful biosynthesis of diosmin, which is used as a medicine, in *N. benthamiana* paves the way for producing other commercially important flavonoids with pharmaceutical properties through a multigene expression system in plants.

## Data Availability

The original contributions presented in the study are included in the article/**Supplementary Material**, further inquiries can be directed to the corresponding author/s.
