# Supplementary material for: Tailored biosynthesis of diosmin through reconstitution of the flavonoid pathway in Nicotiana benthamiana
Source: Front Plant Sci. 2024 Oct 18;15:1464877. doi: 10.3389/fpls.2024.1464877 (PMC11527692; doi:10.3389/fpls.2024.1464877)
Supplement: Supplementary file 1 [file DataSheet1.docx]

***Supplementary Material***

**
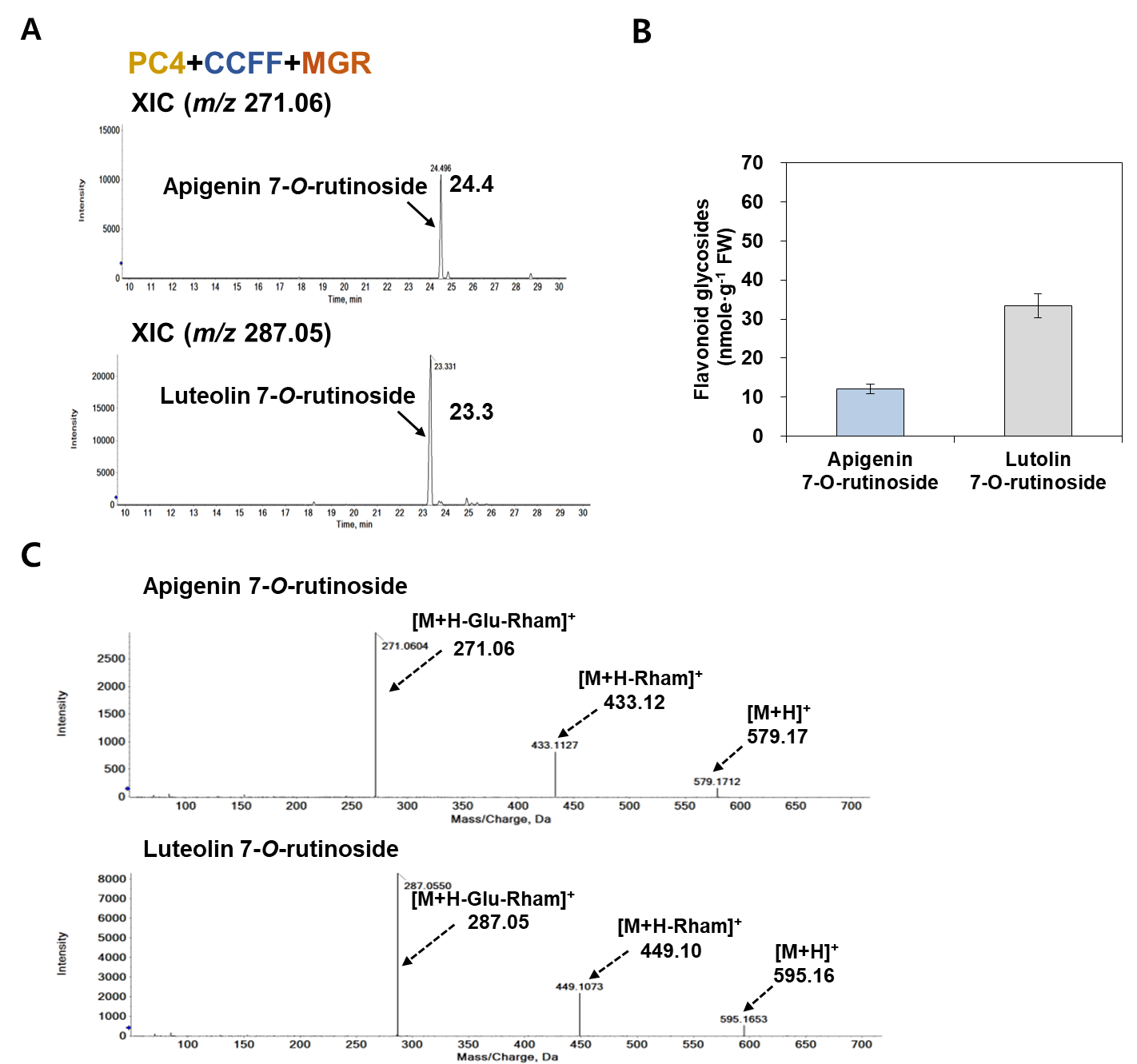
**

**Supplementary Figure 1. Identification of flavone glycosides by transient co-expression of three modules (PC4+CCFF+MGR) in *N. benthamiana.***

*N. benthamiana* leaves were agro-infiltrated with GV3101 strains harboring binary vectors containing multiple genes: PC4 (P, AtPAL; C, AtC4H; 4, Sh4CL), CCFF (C, OsCHS; C, BrCHI; F, OsF3′H; F, OsFNS), and MGR (M, PaF4′OMT; G, CsUGT76F1; R, Cs1,6RhaT). Flavonoid glycosides profiles from the infiltrated leaves were analyzed by UPLC-DAD-QToF/MS in positive ion mode. **(A)** Extracted-ion chromatogram (XIC) at *m/z* 271.06 and *m/z* 287.05 representing protonated apigenin 7-*O*-rutinoside (RT: 24.4min) and luteolin 7-*O*-rutinoside (RT: 23.3min), respectively, in agro-infiltrated leaves. **(B)** Flavone glycoside (apigenin 7-*O*-rutinoside, luteolin 7-*O*-rutinoside) contents in agro-infiltrated leaves. Average apigenin 7-*O*-rutinoside and luteolin 7-*O*-rutinoside contents were calculated based on the areas of the corresponding aglycone standard (apigenin, luteolin), respectively. Mean values ± SE (standard error) of three independent biological samples are shown. **(C)** Representative XICs at *m/z* 271.06 and *m/z* 287.05 showing MS/MS spectra of apigenin 7-*O*-rutinoside and luteolin 7-*O*-rutinoside in infiltrated leaves.

**
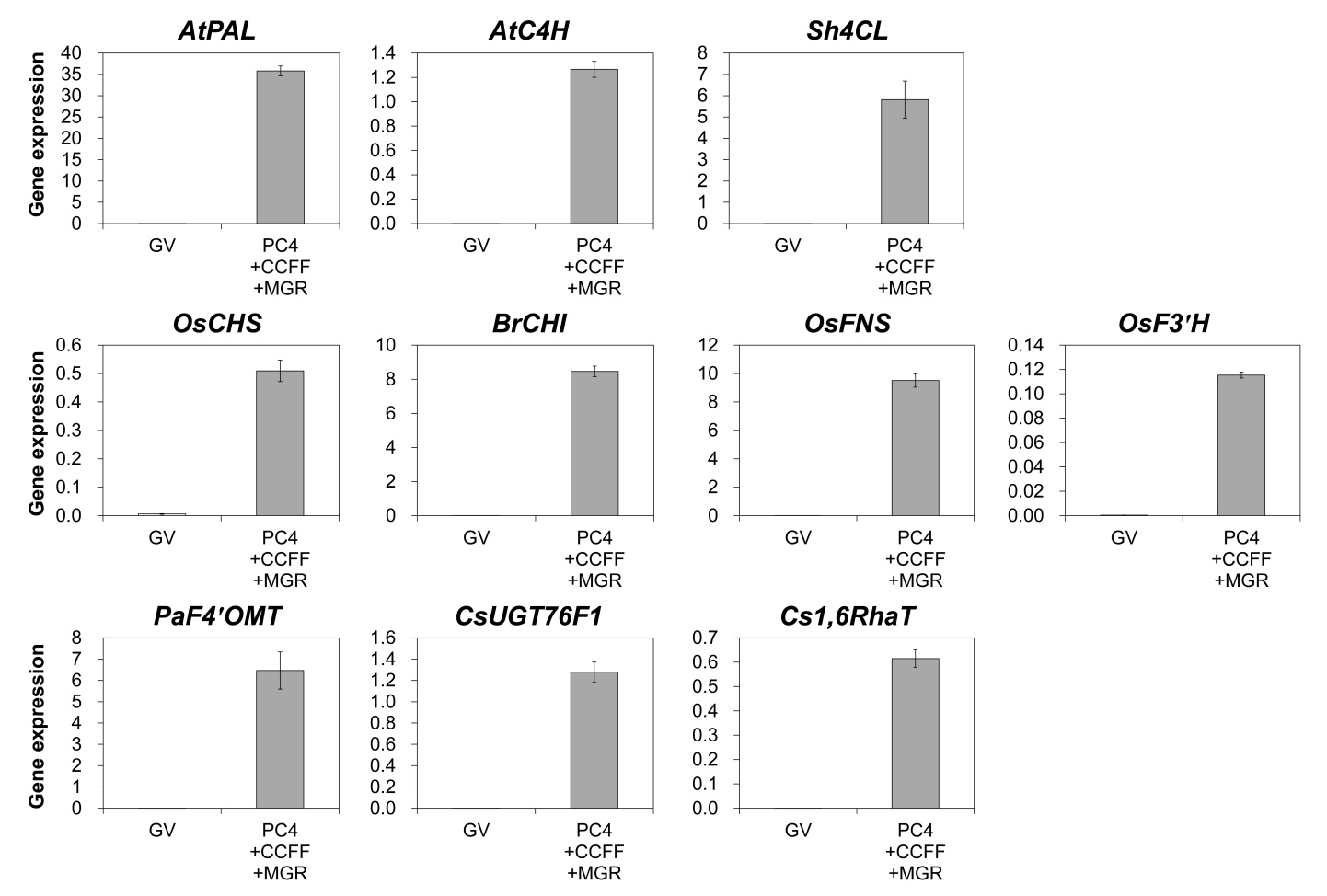
Supplementary Figure 2. Gene expression (2^-∆Ct^) analysis of leaves co-infiltrated with three modules (PC4+CCFF+MGR) in *N. benthamiana.***

*N. benthamiana* leaves were agro-infiltrated with GV3101 strains harboring binary vectors containing multiple genes: PC4 (P, AtPAL; C, AtC4H; 4, Sh4CL), CCFF (C, OsCHS; C, BrCHI; F, OsF3′H; F, OsFNS), and MGR (M, PaF4′OMT; G, CsUGT76F1; R, Cs1,6RhaT). Total RNA was isolated from the agro-infiltrated leaves at 6 days after infiltration and cDNA was synthesized for qRT-PCR anaysis using gene-specific primer sets. *NbPP2A* was used as an internal control. Mean values ± SE (standard error) of three independent biological samples are shown.

**Supplementary Table 1. Accession numbers, plant sources, and PCR primer sequences of genes used for cloning**

| **Gene** | **Accession number** | **Plant source** | **Purpose** | **Primer** | **Primer sequence (5′ to 3′)** |
| --- | --- | --- | --- | --- | --- |
| *AtPAL* | AT2G37040 | *Arabidopsis thaliana* | Level 0 cloning | F | TTGAAGACAAAATGGAGATTAACGGGGCACAC |
|  |  |  |  | R | TTGAAGACAAAAGCCCTTAACATATTGGAATGGGAGCTC |
|  |  |  | qPCR | F | AGCCTACGATAACGGAACATCGGC |
|  |  |  |  | R | GAACTCTTCTCCAGGCGACGTCAC |
| *AtC4H* | AT2G30490 | *Arabidopsis thaliana* | Level 0 cloning | F | TTGAAGACAAAATGGATCTGCTACTCCTTGAGAA |
|  |  |  |  | R | TTGAAGACAAAAGCTTAGCAGTTCCGCGGCTTCATAAC |
|  |  |  | qPCR | F | CCTGAGATTCAGTCCAAGCTTCG |
|  |  |  |  | R | GAGGAACGAGGAGTGGGATAG |
| *Sh4CL* | SRX682844 | *Sinopodophyllum hexandrum* | Level 0 cloning | F | TTGAAGACAAAATGGAGACTCCTCCCCAAGA |
|  |  |  |  | R | TTGAAGACAAAAGCCTAATTAGGAAGATCAGCTGCTAGC |
|  |  |  | qPCR | F | AGGGTTGGTGACTAGTGTGG |
|  |  |  |  | R | CATAATCAAAATCGCCGCGC |
| *OsCHS* | LOC_Os11g32650 | *Oryza sativa* | Level 0 cloning | F | TTGAAGACAAAATGGCTGCTGCAG |
|  |  |  |  | R | TTGAAGACAAAAGCTCATGCCGCG |
|  |  |  | qPCR | F | TTGAAGCTAAGGTCGGCCTTGAC |
|  |  |  |  | R | GCATGACCATCTTCTGCACTACG |
| *BrCHI* | Bra003209 | *Brassica rapa* L. | CDS Cloning | F | ATGTCTTCTTCCAACTGTCCGTC |
|  |  |  |  | R | TCAGTTCTCTTTGGCCAGTTTATC |
|  |  |  | Level 0 cloning | F | TTGAAGACAAAATGTCTTCTTCCAACTGTCCGTC |
|  |  |  |  | R | TTGAAGACAAAAGCTCAGTTCTCTTTGGCCAGTTTATC |
|  |  |  | qPCR | F | CAAGGACGAAACGTTCCCTCC |
|  |  |  |  | R | CAAGAACTGCCTCTGCCAAC |
| *OsFNS* | AK100972 | *Oryza sativa* | Level 0 cloning | F | TTGAAGACAAAATGGCATCATTG |
|  |  |  |  | R | TTGAAGACAAAAGCTCACAGGAG |
|  |  |  | qPCR | F | CATGCCATTTGGATCTGGAAGGAG |
|  |  |  |  | R | CCTAGCACACACCAAACCATCTGC |
| *OsF3'H*  (*OsCYP75B3*) | AK064736.1 | *Oryza sativa* | Level 0 cloning | F | TTGAAGACAAAAGCTCAAACACC |
|  |  |  |  | R | TTGAAGACAAAATGGACGTAGTGC |
|  |  |  | qPCR | F | GAAGAGCAGAAGTTGGACGGTGAC |
|  |  |  |  | R | GTCTAATCAACTCCGCTAGTGCCC |
| *PaF4'OMT* | KY977687 | *Plagiochasma appendiculatum* | pGEX-6P-1  (infusion) | F | GGGGCCCCTGGGATCCATGGCGGTATCCACGAATGG |
|  |  |  |  | R | GGAATTCCGGGGATCCCTACTTCCGGACCTCTATGATGTC |
|  |  |  | Level 0 cloning | F | TTGAAGACAAAATGGCGGTATCCAC |
|  |  |  |  | R | TTGAAGACAAAAGCCTACTTCCGGAC |
| *MpOMT4* | AK337461 | *Mentha* × *piperita* | pGEX-6P-1  (infusion) | F | GGGGCCCCTGGGATCCATGGTGGCGGATGAAGAA |
|  |  |  |  | R | GGAATTCCGGGGATCCTCAAGGATAGGCCTCAATGACA |
|  |  |  | Level 0 cloning | F | TTGAAGACAAAATGGTGGCGGATG |
|  |  |  |  | R | TTGAAGACAAAAGCTCAAGGATAGGCCTC |
| *SOMT2* | TC178411 | *Glycine max* | pGEX-6P-1  (infusion) | F | GGGGCCCCTGGGATCCATGGTTGAGCTTGACATACC |
|  |  |  |  | R | GGAATTCCGGGGATCCTCAAGGATAGATCTCAATAAGAGAC |
| *CreOMT1* | LC507211 | *Citrus reticulata* Yoshida Ponkan | pGEX-6P-1  (infusion) | F | GGGGCCCCTGGGATCCATGAATTTGATCGATGGAGAGC |
|  |  |  |  | R | GGAATTCCGGGGATCCTTAAGGACAAACCTCAATAAGAGA |
| *CreOMT4* | LC507213 | *Citrus reticulata* Yoshida Ponkan | pGEX-6P-1  (Infusion-) | F | GGGGCCCCTGGGATCCATGGATATGAATCATATGATTAATGC |
|  |  |  |  | R | GGAATTCCGGGGATCCTCAGGGGTAAAGCTCAATAAG |
| *CsUGT76F1* | KDO69246 | *Citrus sinensis* | Level 0 cloning | F | TTGAAGACAAAATGGATCAAAGAAAAGGTCGCAG |
|  |  |  |  | R | TTGAAGACAAAAGCTTACTGAGTGTGGAAAGTAAATGTCTC |
|  |  |  | qPCR | F | GTGAGCTTTGGGAGTATTGCAG |
|  |  |  |  | R | CATGAACCCACTTGGCAATGG |
| *Cs1,6RhaT* | DQ119035 | *Citrus sinensis* | pENTR-D-TOPO cloning | F | CACCATGCACGCCCCTTCGAAC |
|  |  |  |  | R | TTAAGCTAAGGCTTTGAGATCCTTG |
|  |  |  | pGEX-6P-1  (infusion) | F | GGGGCCCCTGGGATCCATGCACGCCCCTTCGAAC |
|  |  |  |  | R | GGAATTCCGGGGATCCTTAAGCTAAGGCTTTGAGATCCTTG |
|  |  |  | qPCR | F | GGAAATTACGGGATTGCCCTTC |
|  |  |  |  | R | CATCCTGTGTGCACCACTC |
| *CiRhaT-GD4x* (UGT97A20)/  *CiRhaT-H2x/*  *CiRhaT-HB2x*  (UGT79A22) | OL422134/  OL422136 | *Chrysanthemum indicum* | pENTR-D-TOPO cloning | F | CACCATGTCTATGAATGGAAAAGATAAAG |
|  |  |  |  | R | CTACAGGACGATGTCTTGTGTAAGAG |
|  |  |  | pGEX-6P-1  (infusion) | F | GGGGCCCCTGGGATCCATGTCTATGAATGGAAAAGATAAAG |
|  |  |  |  | R | GGAATTCCGGGGATCCCTACAGGACGATGTCTTGTGTAAGAG |
| *NbPP2A* | TC21939 | *Nicotiana benthamiana* | qPCR | F | GACCCTGATGTTGATGTTCGCT |
|  |  |  |  | R | GAGGGATTTGAAGAGAGATTTC |

**Supplementary Table 2. Parts comprising MoClo level 1 and level M gene expression cassettes**

| **MoClo**  **destination vector** | **Vector ID** | **Promoter** | **Gene** | **Terminator** | **Gene construct** |
| --- | --- | --- | --- | --- | --- |
| Level 1 | P | AtuMas | *AtPAL* | CaMV35S Ter |  |
|  | C | ProCaMV35S Short | *AtC4H* | AtuOCS Ter |  |
|  | 4 | ProCaMV35S Long | *Sh4CL* | Atug7 Ter |  |
|  | C | ProAtUBQ10 | *OsCHS* | Atug7 Ter |  |
|  | C | AtuOcs | *BrCHI* | AtuOCS Ter |  |
|  | F | ProAtRbcS2B | *OsFNS* | AtuNos Ter |  |
|  | F | AtLHB1B1 | *OsF3′H* | AtuMasTer |  |
|  | M | ProCaMV35S Long | *PaF4′OMT* | AutOcs Ter |  |
|  | G | SlRbcS1 | *CsUGT76F1* | Atug7g7 Ter |  |
|  | R | ProCaMV35S Short | *Cs1,6RhaT* | AutMas Ter |  |
| Level M | PCFF |  |  |  | *AtPAL*, *OsCHS*, *OsFNS*, *OsF3′H* |
|  | PC4 |  |  |  | *AtPAL*, *AtC4H*, *Sh4CL* |
|  | CCFF |  |  |  | *OsCHS*, *BrCHI*, *OsFNS*, *OsF3′H* |
|  | MGR |  |  |  | *PaF4′OMT*, *CsUGT76F1*, *Cs1,6RhaT* |

**Supplementary Table 3. Detailed descriptions of promoters and terminators used in MoClo Golden Gate vectors**

| **Abbreviation of**  **promoter and terminator** | **Full name of promoter and terminator** |
| --- | --- |
| AtuMas | Promoter + 5′UTR, mas, (*Agrobacterium tumefaciens*) + 5′UTR |
| ProCaMV35S Short | Promoter (0.4 kb), 35S (Cauliflower Mosaic Virus)  + 5′UTR, Ω (Tobacco Mosaic Virus) |
| ProCaMV35S Long | Promoter (1.3 kb ), 35S (Cauliflower Mosaic Virus)  + 5′UTR Ω (Tobacco Mosaic Virus) |
| ProAtUBQ10 | pPAtUbq10 (GB0223) |
| AtuOcs | Promoter + 5′UTR, ocs, (*Agrobacterium tumefaciens*) + 5′UTR |
| ProAtRbcS2B | Promoter + 5′UTR, RbcS2B (AT5g38420, *Arabidopsis thaliana*) |
| SlRbcS1 | Promoter and 5′UTR, RbcS2 (*Solarnum lycopersicum*) |
| AtLHB1B1 | Promoter, LHB1B1 (AT2g34430, *Arabidopsis thaliana*) |
| CaMV35S Ter | 3′UTR, polyadenylation signal/terminator, 35S (Cauliflower Mosaic Virus) |
| AtuOCS Ter | 3′UTR, polyadenylation signal/terminator, ocs (*Agrobacterium tumefaciens*) |
| Atug7 Ter | 3′UTR, polyadenylation signal/terminator, Atug7 (*Agrobacterium tumefaciens*) |
| AutMas Ter | 3′UTR, polyadenylation signal/terminator, mas (*Agrobacterium tumefaciens*) |
| AtuOCS Ter | 3′UTR, polyadenylation signal/terminator, ocs (*Agrobacterium tumefaciens*) |
| AtuNos Ter | 3′UTR, polyadenylation signal/terminator, nos (*Agrobacterium tumefaciens*) |
